# Navigating wellness through integration: coping strategies for depression among Syrian refugees in Norway

**DOI:** 10.1186/s40359-024-01987-0

**Published:** 2024-09-14

**Authors:** Dixie Brea Larios

**Affiliations:** 1 Department of Psychosocial Science, University of Bergen, Christies gate 12, Postboks 7807, Bergen, 5015 Norway; 2 Research Department at the Oral Health Centre for Expertise in Western Norway, Postbox 2354 Møllendal, Bergen, 5867 Norway

**Keywords:** Integration, Coping with depression, Help-seeking, Refugees, Syrians

## Abstract

**Background:**

Refugees frequently face the challenges of adapting to unfamiliar environments and new cultural contexts. Such adaptations emphasize the importance of coping strategies during resettlement and for individuals to successfully integrate in the new communities. Particularly for Syrian refugees in Norway, many of whom have escaped war and conflict, understanding their ways of coping with mental health problems, such as depression, is pivotal.

**Method:**

This study used a cross-sectional study design to explore the relationship between integration aspects and coping strategies for depression from the Syrian population living in Norway. Syrian participants (*N* = 96) answered questions from the Brief COPE, the Hopkins symptom checklist (HSCL-13), and the Immigration Policy Lab index (IPL-12/24).

**Results:**

Hierarchical multiple regression analysis indicated that employing a problem-focused coping strategy was significantly associated with better psychological and social integration outcomes among Syrian refugees in Norwegian society.

**Conclusion:**

The study suggests that certain aspects of integration, such as feeling a sense of belonging and actively seeking help, significantly improve mental health outcomes for Syrian refugees. Emphasizing problem-focused coping strategies could be vital in facilitating the successful integration of refugees into Norwegian society.

**Supplementary Information:**

The online version contains supplementary material available at 10.1186/s40359-024-01987-0.

## Introduction

In 2023, Norway had 103,606 refugees, mainly from Syria (15%), Eritrea (7%), Afghanistan (3%), and more recently from Ukraine (62%) [1]. Refugees continually encounter risky conditions during flight that may increase their vulnerability to mental health problems. The experience of witnessing war and the motivations for seeking refuge can significantly impact the health of refugees, particularly their mental well-being (e.g.,[2–6]). The stages associated with the migration process, such as pre-departure and flight experiences (e.g., refugee camps, escape routes, detention), are characterized by uncertainty and fear, which can have enduring effects on mental health [7–9]. These traumatic experiences can lead to psychological distress, including depression and post-traumatic stress disorder (PTSD). Research conducted in Turkey, Switzerland, and Germany has highlighted the influence of stressors and prior trauma on the mental health of Syrians in the aftermath of war and conflict (e.g., [10–14]). Furthermore, unaccompanied minors have also shown high rates of post-traumatic stress disorder (PTSD), depression, and anxiety symptoms shortly after arrival [15–20]. Post-migration circumstances such as language and cultural barriers, economic insecurity, inadequate healthcare, and insufficient social services, can also cause significant stress and anxiety for Syrian refugees, impacting asylum application processes in Norway [21–26]. Often these experiences arise from limited access to essential services such as healthcare, education, employment, and social support networks in the host countries (e.g., [27–29]). All of these can lead to feelings of isolation and anxiety, which can further exacerbate existing mental health concerns (e.g., [8, 30–33]). In recent systematic reviews [7, 34, 35], these aspects and the need for culture-sensitive interventions have been highlighted as key aspects for overcoming the current challenges and meeting the needs of refugees.

### Migration and coping with mental health challenges

The migration process may also involve adjusting to unfamiliar environments and cultural situations, increasing the emphasis on coping strategies and quality of life during resettlement for these refugee populations. Whether it is war and conflict, migration, or its impact on mental health, there is importance in understanding how refugees cope with difficult situations, particularly when suffering a mental health problem while integrating in the society. How to cope with specific stressors among the refugee population in Norway has not yet been explored. Given these aspects, knowledge of how to cope with mental health problems among refugee groups is essential. This study aims to explore coping strategies for depression among Syrian refugees during the integration process in Norway. The hypothesis proposed that coping with depression might be associated with various aspects of integration, as suggested by Harder and colleagues [36]. Understanding the psychological impact of migration, support interventions can be designed to make the transition smoother and aid refugees in adjusting to their new environment. This transition can include social and psychological support, access to medical care, and other resources. Actively engaging in community support networks and drawing upon certain ways of coping during the integration process may indicate better mental health outcomes, including reduced symptoms of depression [14, 29]. Developing such coping strategies is crucial for migrants, especially refugees, to successfully integrate.

### Coping strategies and integration

For a better understanding of how successful integration is measured, Harder and colleagues [36] have first defined integration as the “degree to which immigrants have the knowledge and capacity to build a fulfilling, successful life in the host society” [36]. This definition encompasses several aspects of integration, capturing the essence of all migrant group situations in terms of knowledge (e.g., the ability to navigate the host country and fluency in the national language) as well as capacity (aware of the need to invest in the resettlement country with mental social and economic resources).

In many resettlement countries, the successful integration of refugee groups is crucial, especially when making effective social policies. An Introduction Program in Norway (www.regjeringen.no; www.imdi.no) provides rights and mandatory participation for newly arrived refugees and those granted asylum to obtain basic qualifications. Municipalities adapt the program, emphasizing social studies, education, the Norwegian language, and on-the-job training to increase social life and labor market inclusion. Such programs are relevant for Syrian refugees' successful integration and the development of coping strategies. In the long run, language learning varied between groups; women experienced a greater impact on income earnings, and there were negative effects for those participants who had children in another country than Norway when they began working [37, 38]. These findings may compromise some aspects of future integration for these groups.

Harder and colleagues [36] developed six dimensions to identify successfulintegration. These include the psychological (e.g., feelings of connection), social (e.g., social interactions), navigational (e.g., acquiring basic needs), economic (e.g., income), linguistic (e.g., understanding the dominant language), and political integration (e.g., understanding the political issues of the host country) [39]. These dimensions are intended to measure integration among migrant groups (including refugees and asylum seekers) and not the native population. It is important to note that integration is an ongoing process, not a one-time event. These measures of integration focus on the capacity and knowledge migrant groups adapt to a new culture and build a successful life without losing their cultural heritage [36]. Other integration models are consistent with this standpoint [40–43].

Integration can also be seen as a function of coping with stressors when integrating in the host- society, influencing the acculturation aspect [43]. Different ways of coping may reduce acculturative stress, such as creating social networks, building an identity, and maintaining cultural practices, enhancing the integration process. Developing coping skills can help refugees adjust to their new environment and build a successful life that honors their cultural heritage, maximize their potential in the new society, and develop a sense of belonging. Therefore, measuring the capacity to learn a language, participate in the labor market, and access public services, among others, are key components of successful integration.

According to various coping models [19, 44–46], individuals use different strategies to cope with stress depending on the situation and their personal characteristics. The notion that coping patterns can be malleable has also been explored previously [47–49]. In the words of Lazarus and Folkman (1984), coping involves “constantly changing cognitive and behavioral efforts to manage specific external or internal demands that are appraised as taxing or exceeding the resources of a person” (p.141). Coping can also be classified as problem-focused (managing external demands) and emotion-focused (managing own emotional responses) to reduce stress caused by adverse experiences [47, 48, 50]. Individuals can benefit from certain strategies to manage stressors better, especially with environmental changes such as resettling into a new country, if they recognize and use their malleable coping patterns (e.g., positive thinking, problem-solving skills, setting realistic goals, mindfulness, and relaxation techniques). As a result of a situational context and desired outcomes, coping strategies can also be adaptive or maladaptive [51–53]. By identifying and modifying their coping strategies, refugees can be better equipped to manage these stressors and integrate into their new environment more successfully. This paper wishes to assess this by focusing on how coping strategies for depression can affect the process of integration in the Syrian population.

### Challenges to integration

For the Syrian population resettled in other countries, the process of integration is seen in different aspects such as education, social contact, and labor market activities [54–57]. In the years following the 2015 Syrian crisis, the refugee population has changed. The Syrian population has had significant mental health needs regarding quality of life, health equity, and integration into Norwegian society [58–62]. Their explanatory models of mental health problems have been shown to include transformations when identifying preferred treatment and help-seeking strategies [63]. Integration aspects from a social and psychological perspective require an understanding of the perceived barriers encountered during the migration process, as well as the dynamics that may affect their health [64–66], such as failure to cope with depression and stressful situations. The success of integrating into the host society may depend on the motivation of the population group and their personal commitment to the integration process. The migration process can, however, vary according to other aspects. Refugees in Norway, for instance, may face different situations and requirements compared to other migrant groups because of their refugee status or asylum seeker application processes [67]. It is also important to consider the power dynamics between the host and migrant communities and the national policies that enable and promote integration. Without the right laws, policies, and social infrastructure, integration can be difficult and often fails to create a cohesive society, leading to aspects that may affect the migrant process or, in severe cases, the mental health of individuals. Therefore, it is vital to understand these coping strategies as they may have implications for certain integration policies and community support. Even though refugees may be more affected by war and conflict than other migrant groups and the general population [68], changes in health due to the process of migration and the connections to their home countries (family and friends) persist long after flight and resettlement [16, 45, 69, 70]. Consequently, these connections can have a positive effect on the integration process, providing refugees with a sense of hope and a support network, as well as mitigating the mental health issues related to displacement. Therefore, understanding how Syrian refugees cope with stressful situations and mental health problems is essential to avoid the adverse aspects of the migration process.

For integration to be successful, the host and migrant communities must have equal access to resources and opportunities, such as the provision of educational opportunities to enter the labor market (e.g., introduction programs and access to higher education) [71]. Successful integration is much more difficult without the necessary resources and infrastructure. When refugees have been affected by traumatic situations and mental health problems such as depression, they can be a major barrier to successful integration as they can lead to feelings of isolation, powerlessness, and mistrust, which is why coping strategies are regarded as essential for integration. Understanding the ways of coping among refugee groups and investing in the resources needed to provide mental health support services, such as counseling and follow-up programs, can be an essential part of the integration process, enabling refugees to overcome these issues and feel more connected to their new communities. By exploring the association between integration patterns and depression coping strategies, this study aims to identify the underlying patterns of integration in the Syrian population in Norway.

## Method

### Participant characteristics

This study was conducted as part of a broad online survey investigation targeted at refugee groups living in Norway. The data collected for this study came from the Syrian sample. Ninety-six participants from Syria (40% females) aged 18 to 70 participated in this study.

### Sampling procedure

The data collection from the Syrian sample was conducted between the fall of 2019 and the spring of 2020 before the pandemic of Covid-19. The Norwegian Agency for Shared Services in Education and Research (SIKT—formerly NSD), notification form: 602,214 approved the survey. The study used a cross-sectional design consisting of scale instruments with 104 items from a broader survey investigation. This study assessed the participants' socio-demographic data and coping strategies for depression with different scales of aspects of integration from the primary survey.

The survey was distributed to potential Syrian respondents (*N* = 264) living in Norway over the age of 18, with *N* = 199 (75%) participants consenting and responding to the primary survey. Participants were reached out through the introduction and adult educational programs held in Norway’s three largest cities (Oslo, Bergen, and Trondheim). To raise awareness of the topic and the importance of this study, several presentations were made to potential participants in different communities (Vestland, Agder, and Trøndelag counties). The survey was conducted onsite through iPads and mobile devices provided by the research team. Participants had the option to complete the survey in Arabic or Norwegian, or paper format. An Arabic research assistant was onsite to provide support. Informed consent was obtained from all participants before answering the survey. Participants received no compensation for participation. The research team also distributed flyers and sent e-mail invitations containing the survey link to various Norwegian institutions in cities that work with refugees.

After answering the demographic characteristics, the survey was randomized to one of two embedded surveys. Ninety-three participants dropped out before randomization. This study included only participants from Syria. Norwegian-born participants and incomplete data were excluded. For this study, *N* = 96 (48%) participants responded to the Brief COPE scale with one missing data point. No data was stored for specific variables or participants.

The survey was translated into Norwegian and Arabic languages by a professional translation agency in Norway (e.g., Semantix). The research team and native speakers conducted the back-translation of the language used in the survey. The translations were revised, compared with the original standardized versions, and corrected.

### Dimensions of integration

#### Immigration policy lab

To explore integration, the Immigration Policy Lab index (IPL-12/24) was used, which is a questionnaire that assesses six aspects of integration: *psychological, linguistic, economic, social, navigational,* and *political* [36, 39]. For the aim of this study, the political dimension of integration was considered less relevant and had no clear link to coping strategies with depression. Therefore, the questions for this dimension were not included. The IPL-12/24 index has been validated in international studies conducted in migrant populations [36, 72–74]. The scale used in this study included ten items following the IPL-12 measures described in Harder’s supplementary material. Additional items from the psychological and the social dimensions of integration were included from the IPL-24, consisting of interpersonal factors, awareness of general proprieties, and the ability to handle basic requirements in Norway [39].

#### Psychological integration

The psychological dimension of integration included four items capturing the respondents' feelings of connection with Norway, their wish to continue living there, and their sense of belonging in the host country (e.g., "How connected do you feel with Norway?”, scoring from *I do not feel a connection at all* (1) to *I feel an extremely close connection* (5)). The test of internal consistency yielded a Cronbach alpha of 0.83 and McDonald’s omega of 0.83.

#### Social integration

The social dimension sought to capture the social interactions of the participants. Three items from the IPL-24 were included in the analysis (e.g., "In the last 12 months, how often did you eat dinner with Norwegians who are not part of your family?"). From *never* (1) to *almost every day* (5). Both Cronbach alpha and McDonald’s omega were low (0.65) and (0.68) respectively, but given the purpose of the study, still deemed to be adequate.

#### Linguistic integration

The linguistic dimension of integration has four components of English communication (i.e., reading, listening, writing, and speaking) in the IPL-24 scale. The survey for this study only used two items to capture the ability to speak and read the Norwegian language. The two items included were: “I can read and understand the main points in simple newspaper articles on familiar subjects” and “In a conversation, I can speak about familiar topics and express personal opinions.” From *very well* (5) to *not well at all* (1).

### Measurement of coping

The primary survey contained items for coping behaviors from different scales to measure the coping strategies [47, 75, 76]. This study included the measures of coping strategies from the 28 items of the Brief Cope scale.

#### Brief COPE

To assess the coping strategies, this study used the shortened version of the original 60-question Coping Orientation to Problems Experienced Inventory (COPE), called Brief COPE [47, 77]. Brief COPE uses 28 items to measure how individuals cope with stressful life events and difficult problems. The Brief COPE scale is organized into 14 types identified by Carver [77] (each consisting of two items per type). Brief COPE has been translated into several languages [78–83] and has been validated in different populations and countries [77, 84, 85]. With a Cronbach's alpha of 0.83 [47, 77], different approaches have also been described and reported in previous literature [86–88]. The test of internal consistency yielded a Cronbach's alpha coefficient of 0.75 (0.76 males and 0.75 females) for all items of the Brief Cope [89].

This study followed Carver’s [77] assessment for the Brief Cope scale, which has been divided into three main coping strategies (problem-focused, emotion-focused, and avoidance), each of which included specific characteristics for the 14 types of coping. The problem-focused coping included characteristics of active coping, instrumental support, planning, and positive reframing with sample items such as “I have been taking action to try to make the situation better” and “I have been getting help and advice from other people.” The emotion-focused coping is characterized by venting, emotional support, humor, acceptance, self-blame, and religion facets, with sample items: “I have been learning to live with it” and “I have been praying or meditating.” The avoidant coping included self-distraction, denial, substance use, and behavioral disengagement characteristics with sample items: “I have been saying to myself 'This is not real'” and “I have been using alcohol or other drugs to make myself feel better.” As a result of these characteristics, we were able to gain an understanding of the Syrian population's coping strategies for depression. The responses were on a 4-Likert scale, ranging from (1) I have not been doing this at all to (4) I have been doing this a lot.

### Assessment of depression

The depression assessment consisted of a vignette with a male and a female character exhibiting symptoms of depression according to ICD-10 [90] that has been developed in previous studies on migrants in Norway [91, 92]. The vignette was adapted to the study population using a traditional Syrian male or female name to facilitate identification. After reading the vignette, participants were asked to select the best alternative for to what extent they would have acted if they had been the vignette character using the Brief Cope scale.

#### Hopkins symptoms checklist (HSCL)

Depression was also ascertained with the Hopkins symptoms checklist (HSCL-25) [93]. HSCL-25 is a widely used instrument that assesses depression and anxiety and is mainly used in different settings and population-based studies [94]. The HSCL-25 consists of thirteen items for depression, two for somatic symptoms, and ten for anxiety. Following the assessment of depression with the depression vignette, this study only used a subscale with the 13 items of depressive symptoms from the HSCL-25 (Feeling no interest in things; Feeling everything is an effort; Loss of sexual interest or pleasure; Feeling blue; Worrying too much about things; Crying easily; Feeling hopeless about the future; Feelings of worthlessness; Blaming oneself for things; Thoughts of ending one’s life; Feeling lonely; Feeling trapped or caught; and Feeling low in energy, slowed down). The depression subscale is a good instrument for symptoms of early depression among refugees. The period reference for the current study was “in the last week” when the questionnaire was taken. Each item was rated from (1) not at all to (4) extremely. The HSCL-25 has been previously used in different studies on migrant groups, including refugees [94–97]. A cut-off score of 1.75 has been previously used to identify clinically significant symptoms [98]. However, some studies have suggested that neither the usual cut-off nor the instrument as a whole might be appropriate for certain populations [98–100]. The current study was careful in setting a clinical cut-off even though many of the respondents had reported depressive symptoms.

### Sociodemographic data

The survey included sociodemographic questions about age (collected in ten-year brackets), gender (males and females), and education level (1 = Have not completed any education, 2 = Primary school, 3 = secondary school, 4 = Secondary or university education), which are included in this study as potential covariates.

### Statistical analysis

The primary survey was created using the SurveyXact software program (www.surveyxact.no). A data set and a report were exported from the software program to manage the data in SPSS statistical software 2.0 [89]. Preliminary analyses and intercorrelations were conducted between all the variables for this study. The study relied on the item-subscale scores (13 items for depression) from the individual responses on the HSCL-25 instead of the full 25 items [101]. We tested the normality of assumptions and found one missing data from the coping variable scale *N* = 95. Next, a hierarchical regression analysis was assessed to examine the ability of different coping strategies variables for depression in predicting integration after controlling for sociodemographics. Scores on the HSCL-13, the problem-focused, emotion-focused, and avoidant coping variables were included as predictors in the analysis. We used a sensitivity power analysis to detect a minimum effect based on our sample size (G*Power Version 3.1.9.6). A sample of 96 participants would be sensitive to the effects above Cohen's d = 0.3 with 80% power (alpha = 0.05, two-tailed) [102, 103].

## Results

### Descriptive characteristics

A total number of *N* = 96 participants from Syria (40%females) completed the questionnaire with the Brief Cope scales. The age of the participants ranged from 18 to 70 years with a mean of 3 (SD = 1.062) (in ten-year brackets from 1 to 7). Over half of the participants were between the ages of 20 and 39; most were married, had children, and lived in a large city (19.2%) (see Table 1).

**Table 1:** Sociodemographic characteristics of the population sample

Sociodemographic	
Characteristics	n %
Gender^a^	96
Males	58 60%
Females	38 40%
Age^b^	2.86 (1.062)
Age upon arrival in Norway^c^	4.37 (1.174)
Marital status	1.34 (.475)
Parent	1.45 (.499)
Level of education^d^	3.11 (.988)
Have not completed any education	11 2.4%
Primary school	43 9.2%
Secondary school	36 7.7%
Secondary or university education	84 18%
Current life situation^e^	3.14 (2.371)
In paid work	46 10%
In school	52 11%
Unemployed (actively/not actively looking for a job)	42 9.0%
Sick	5 1.1%
Other	31 6.6%
Living area	2.15 (1.329)
Large city	90 19.2%
Outskirts or suburb	16 3.4%
Small or medium-sized city	43 9.2%
Village center	17 3.6%
Sparsely populated area	13 2.8%

*N *96

^a^Gender was coded Males (1), Females (2)

^b^Age was collected in ten-year brackets 1–7 (1 = 18–29; 2 = 30–39; 3 = 40–49; 4 = 50–59; 5 = 60–69; 7 = 70 +). M/SD. Min.1 – Max.6

^c^Age upon arrival was collected in ten-year brackets (1–7) Min.1 – Max.6

^d^Level of education was collected in different alternatives recoded in 4 items. Min.1 – Max.4

^e^Other (Permanently sick or disabled, retired, unpaid work, looking after children or other persons)

### Integration, depression, and coping strategies

The relationship between the dimensions of integration (psychological, social, and linguistic), the coping strategies (problem-focused, emotion-focused, and avoidant from the Carver assessment), and depression was investigated using Pearson correlation coefficient. Preliminary analyses were performed to ensure no violation of the assumptions of normality and linearity. There was a strong positive correlation between most of the variables, r = 0.58, *n* = 96, *p* < 0.001, with high levels of the dimensions of integration associated with coping strategies and depression. The psychological dimension of integration negatively correlated with education (r = -0.20) and depression (r = -0.38). See Table 2.

**Table 2:** Descriptive statistics and Correlations for Study Variables

Variable	N	M	SD	1	2	3	4	5	6	7	8	9	10
1. Age	237	2.86	1.06	1									
2.Gender	237	1.37	.484	-.161*	1								
3. Education	174	3.11	.988	-.010	-.004	1							
4. Depression	178	26.8	10.0	-.098	-.108	.032	1						
5. Psycho-logical integration	179	3.13	.888	.176*	-.011	-.203**	-.381**	1					
6. Social integration	175	1.98	.812	.127	-.019	.061	-.169*	.374**	1				
7. Linguistic integration	174	3.04	1.07	-.284**	-.113	.337**	-.074	-.018	.180*	1			
8. Problem Focused	95	23.4	3.77	.041	.048	.052	-.261*	.414**	.269*	-.013	1		
9. Emotion Focused	96	27.2	4.71	.023	-.010	-.140	.089	.068	.126	-.050	.323**	1	
10. Avoidant	96	14.9	2.46	.041	-.239*	-.148	.039	-.028	-.038	.010	.224*	.395**	1

^*^p < .05. **p < .01

### Regression analysis

Hierarchical multiple regression analysis was used to assess three main coping strategies for depression (Problem-focused, emotion-focused, and avoidant coping) to predict the aspects of integration (psychological, social, linguistic) after controlling for the socio-demographic variables. The three main coping strategies were separated into three models (see Tables 3, 4, and 5). Preliminary analyses were conducted to ensure no violation of normality, linearity, homoscedasticity, and multicollinearity assumptions. No violations were detected.

**Table 3:** Hierarchical Regression Results for Psychological Integration

Variable	**B**	**95%CI for B**		**SE B**	**β**	**R**^2^	**△R**^2^
		LL	UL				
Step 1						.072	.072**
Constant	3.231	2.237	4.225	.500	.177		
Age	.148	-.027	.323	.088	.016		
Gender	.030	-.354	.413	.193	-.201		
Education	-.180	-.366	.005	.093			
Step 2						.201	.164
Constant	4.289	3.203	5.376	.546			
Age	.112	-.053	.276	.083	.134		
Gender	-.055	-.416	.306	.181	-.030		
Education	-.170	-.344	.003	.087	-.190		
Depression	-.032	-.050	-.015	.009	-.365		
Step 3						.338	.282
Constant	3.047	1.277	4.818	.890			
Age	.106	-.047.	.258	.077	.127		
Gender	-.143	-.489.	.204	.174	-.078		
Education	-.215	-.380.	-.050	.083	-.239		
Depression	-.023	-.040.	-.006	.009	-.258***		
Problem-focused coping	-.093	.046.	.141	.024	.397***		
Emotion-focused coping	-.002	-.041.	.037	.020	-.011		
Avoidant coping	-.058	-.132.	.015	.037	-.162		

*CI *Confidence interval, *LL *lower limit, *UL *upper limit

*p< .05. **p< .01. ***p< .001

**Table 4:** Hierarchical regression results for social integration

Variable	B	95%CI for B		SE B	β	R^2^	△R^2^
		LL	UL				
Step 1						.020	.020**
Constant	1.540	.605	2.474	.470			
Age	.098	-.066	.262	.083	.128		
Gender	.003	-.357	.364	.181	.002		
Education	.052	-.123	.226	.088	.063		
Step 2						.046	.026**
Constant	1.971	.884	3.058	.547			
Age	.083	-.081	.248	.083	.109		
Gender	-.031	-.392	.329	.181	-.019		
Education	.056	-.118	.229	.087	.068		
Depression	-.013	-.031	.004	.009	-.163		
Step 3						.119	.073**
Constant	.996	-.874	2.865	.940			
Age	.077	-.084	.238	.081	.101		
Gender	-.099	-.465	.267	.184	-.059		
Education	.041	-.133	.215	.088	.050		
Depression	-.009	-.027	.009	.009	-.113		
Problem-focused coping	.049	-.001	.062	.025	.228***		
Emotion-focused coping	.021	-.126	.030	.021	.124		
Avoidant coping	-.048			.039	-.125		

*CI* Confidence interval, *LL* lower limit, *UL* upper limit.

*p< .05. **p< .01. ***p< .001

**Table 5:** Hierarchical regression results for linguistic integration

Variable	B	95%CI for B		SE B	β	R^2^	△R^2^
		LL	UL				
Step 1						.218	.218
Constant	3.289	2.189	4.389	.553			
Age	-.309	-.503	-.116	.097	-.307		
Gender	-.356	-.780	.069	.213	-.161		
Education	-.361	.156	.567	.103	.334		
Step 2						.236	.018**
Constant	3.764	2.482	5.045	.645			
Age	-.325	-.519	-.131	.097	-.323		
Gender	-.394	-.819	.032	.214	-.178		
Education	-.366	.162	.570	.103	.338		
Depression	-.015	-.035	.006	.010	-.136		
Step 3						.241	.005*
Constant	3.739	1.453	6.025	1.149			
Age	-.325	-.522	-.128	.099	-.322***		
Gender	-.365	-.812	.083	.225	-.165		
Education	.382	-.169	.595	.107	.352***		
Depression	-.017	-.039	.005	.011	-.157		
Problem-focused coping	-.020	-.081	.042	.031	-.069		
Emotion-focused coping	.005	-.045	.055	.025	.022		
Avoidant coping	.021	-.074	.177	.048	.049		

*CI* Confidence interval, *LL* lower limit, *UL* upper limit

*p < .05, **p< .01, ***p< .001

For the psychological dimension of integration, the socio-demographic variables age, gender, and education were entered at Step 1, explaining 7.2% of the variance in psychological integration. After the entry of the depression subscale at Step 2, the total variance explained by the model was 20%, *F* (1,85) = 5.36, *p* < 0.001. The problem-focused, emotion-focused, and avoidant coping strategies were entered at Step 3, and the total variance explained by the model was 34%, *F* (3,82) = 5.98, *p* < 0.001. The coping strategy predictors explained an additional 14% variance in the psychological dimension of integration, *R* squared change = 0.14, *F* change (3,82) = 5.654, *p* < 0.001. In the final model, only two predictors were statistically significant, with problem-focused coping recording a higher semi-partial correlation value (*sr* = -0.351, *p* < 0.001) than the depression scale (s*r* = -0.241) (see Table 3).

For the social dimension of integration, the socio-demographic variables age, gender, and education were entered at Step 1, explaining 2% of the variance in social integration. After the entry of the depression subscale at Step 2, the total variance explained by the model was 5%, *F* (1,85) = 1.021, *p* < 0.001. The problem-focused, emotion-focused, and avoidant coping strategies were entered at Step 3, and the total variance explained by the model was 12%, *F* (3,82) = 1.577, *p* < 0.001. The coping strategy predictors explained an additional 7.3% variance in the psychological dimension of integration, *R* squared change = 0.073, *F* change (3,82) = 2.257, *p* < 0.001. In the final model, only one predictor was statistically significant, with problem-focused coping recording a semi-partial correlation value (*sr* = 0.210, *p* < 0.001) compared to the other predictors (see Table 4).

For the linguistic dimension of integration, the socio-demographic variables age, gender, and education were entered at Step 1, explaining 22% of the variance in linguistic integration. After the entry of the depression subscale at Step 2, the total variance explained by the model was 24%, *F* (1,85) = 6.560, *p* < 0.001. The problem-focused, emotion-focused, and avoidant coping strategies were entered at Step 3, and the total variance explained by the model was 24.1%, *F* (3,82) = 3.720, *p* < 0.001. The coping strategy predictors explained an additional 0.5% variance in the psychological dimension of integration, *R* squared change = 0.005, *F* change (3,82) = 0.186, *p* < 0.001. In the final model, only two predictors were statistically significant, with age recording a semi-partial correlation value (*sr* = -0.316, *p* < 0.001) compared to education (*sr* = 0.343* p* < 0.001) (see Table 5).

## Discussion

This study explored the relationship between aspects of integration and coping strategies for depression among the Syrian population living in Norway. The study also wished to determine whether depression symptoms and coping strategies were predictive of successful integration. Our findings suggest that while integrating into Norwegian society, certain demographic factors such as higher educational attainment and age, as well as problem-focused behaviors, play a noteworthy role among Syrian participants in coping with depression.

The study identified three main aspects of successful integration: psychological, social, and linguistic, based on Harder and colleagues' [36] definition. These three aspects were essential for achieving successful integration among the Syrian participants. The importance of psychological well-being for establishing relationships with others, social connections for feeling a sense of belonging, and linguistic skills for effective communication cannot be overstated. Three main coping strategies were identified involving the 14 types from the Brief Cope scale: problem-focused, emotion-focused, and avoidant, each with their specific characteristics. Adaptive coping strategies help foster psychological well-being, nurture social relationships, and enhance linguistic development, all of which are beneficial when adjusting to a new environment.

### Coping strategies and the dimensions of integration

The psychological and social dimensions of integration were associated with problem-focused behaviors such as planning, active coping, positive reframing, and instrumental support. Feelings of connectedness and belonging in Norway are psychological components of integration, and contacting Norwegians, such as talking and having dinner with them, were associated with the social components of integration. There is a possibility that these behaviors are more likely to appear after arrival. For instance, refugees, as newcomers resettling in Norway, may feel overwhelmed when encountering new traditions, rules, and language. By engaging in problem-focused behaviors, refugee groups can create a sense of control and mastery over their environment, which can help them to adjust better and feel connected to the new society. Furthermore, connecting with Norwegians can help create a sense of belonging, which may help to further facilitate integration. Holding a positive attitude, having high expectations for future resettlement, and getting the necessary help from the local community may promote successful integration.

The results on problem-focused coping, such as taking action to improve the situation or getting help and advice from others, could explain the social aspect of integration to actively acquire Norwegian friends and reach out to spend time with them. Furthermore, these behaviors may be strengthened when refugees engage in activities and programs that promote integration, such as language classes and cultural events. This significant contribution could also be related to the linguistic aspect of integration such as learning the Norwegian language and activity participation.

The results also indicated a stronger association between age, education, and the linguistic dimension of integration. For instance, being a young highly educated Syrian may confer a distinct advantage, playing a pivotal role in adjusting to and integrating into Norwegian society. Research has suggested that younger adults and those with higher levels of educational attainment may demonstrate enhanced language acquisition and integrate faster in new environments (e.g., [104, 105]), highlighting that language is a critical factor, but not the only one contributing to integration. For instance, being a young highly educated Syrian may confer a distinct advantage, playing a pivotal role in adjusting to and integrating into Norwegian society. Age and education can serve as determinants in assisting refugees to integrate in their new country. Thus, age and education appear to be the most influential factors in the Norwegian integration process, influencing the speed of language learning and overall integration.

### Depression and successful integration

The results have also indicated that depression also contributed significantly to the psychological dimension of integration. Previous studies have indicated that among those with refugee background have reported high levels of mental health problems [106, 107]. A high prevalence of mental health problems in the Syrian population is also shown, with estimated depression at 40.9% [108, 109]. This prevalence is consistent with the latest studies done in Norway; prevalence of depression among the Syrian population is around 45.2% [109]. Those suffering from depression may have difficulty establishing a sense of belonging and connecting with the Norwegian society (e.g., feeling isolated and an outsider), hindering integration.

The results suggest that Syrians may actively focus on the current situation and seek help despite depressive symptoms; likely because they might be motivated to build a successful life in Norway after the journey, and take active steps to do so, such as seeking help for their depression. However, the regression model showed a small R-square change of 0,5%, indicating less variability than expected in the outcome, and enough data may have explained the proportion of the variance and strength effect. Consequently, the problem-focused coping strategy adopted by Syrians in Norway might be useful in helping them manage depression and other mental health issues, allowing them to integrate into Norwegian society more effectively. This active focus on the situation may enable individuals to overcome their depressive symptoms, establish a sense of belonging, and aid in the integration process.

### Implications

Some of these findings are consistent with previous studies regarding communication and social isolation affecting particularly, the integration of refugees in the host societies (e.g., [14, 110–113]). Healthcare services and coping behaviors may be affected by several parameters; for instance, the Norwegian healthcare system and society may face challenges in meeting the health needs of newly arrived migrants, especially refugees suffering from depression [114]. Syrians may benefit only from problem-focused coping strategies when integrating in Norway, but other refugee groups may experience the opposite, resulting in unfavorable coping strategies that lead to mental health problems going untreated or being overlooked. The integration of refugee groups into Norwegian society could be further complicated by the need to find new sources of support and guidance. Healthcare providers need to be aware of the different situations refugees may face to provide adequate support and resources to address these issues and contribute to successful integration. For the integration and development of coping strategies among refugee populations living in Norway, policies that offer more help and guidance to healthcare providers could be helpful. Future guidelines could emphasize educating the refugee population on identifying and addressing mental health problems in addition to the provision of interpreters, support groups, and mental health services. Providing the necessary resources to the refugee population is essential for their successful integration into Norwegian society.

### Limitations

The current study has some limitations. The results from the cross-sectional study hinder the drawing of causal conclusions, and the self-reported data from the coping and depression scales make it difficult to determine the severity of the symptoms. The HSCL-25 [93] is widely used to both clinical and epidemiological purposes to measure psychological distress. The depression subscale for the Hopkins Symptoms Checklist is used as a screening instrument to assess depression and does not serve as a diagnostic tool. In studies among migrant populations, using HSCL-13 is a suitable measure for this study population and the results gathered with the integration aspects may be useful for further clinical work and research exploration [98]. This study captured the dynamic of the integration process and the other scales at one point in time. As such the design did not capture the dynamics of adapting coping behavior over time. For example, the Syrian population might modify their coping styles over time when faced with another stressful situation. A more extensive interaction analysis, such as using a structural equation model, could have provided a deeper understanding of the relationship between integration and depression. However, the interaction could only be beneficial if two populations were explored and compared. Due to the low response rate and the use of a single population, the study could not achieve this type of interaction. Hence, multiple regression analysis was performed for the selected and used population group. Inconsistencies in the results may be related to current situations or vicarious experiences, such as emotion-focused and avoidant coping. Data collection was closed just before the COVID-19 pandemic began, limiting the sample size, and making implementation, sample availability, and the recruitment of hard-to-reach potential participants challenging [115, 116]. While qualitative interviews were conducted among Syrian refugees prior this study [63], a larger sample size or a follow-up study may improve the reliability of the results. The lack of social support could suggest a lower predictor of self-efficacy [117, 118], raising questions about the effectiveness of coping strategies for depression. However, these predictors could also be influenced by the presence of mental health problems, other traditional values, or external factor affecting the study population. More in-depth research into the impact of cultural values on coping strategies for mental health problems may be needed in larger samples of refugee groups to identify similar patterns within refugee populations.

## Conclusion

Using specific coping strategies for depression could explain why some people are more successful at certain types of coping than others. Depression sufferers can be negatively affected when difficulties arise in accessing quality mental health resources or experiencing increased stress due to acculturation. The lack of social support and needed help may prevent them from effectively implementing the strategies and achieving the desired outcomes. The results of this study have also indicated that depression is associated with the psychological aspect of integration among Syrians, and how age and education positively influence language learning. In light of this, problem-focused coping behaviors are crucial in explaining integration. Consequently, the coping strategies may also be adapted and changed after integration. These changes may also involve the importance of providing support and the necessary resources to refugee groups and help them cope with their new realities. Future studies may investigate further ways of coping through the lens of other groups and peer institutions. Coping with stressful situations may be influenced by new surroundings. By providing support, fostering motivation and empowering with resources to help refugees cope with flight challenges can positively impact their quality of life. Coping strategies are crucial for refugee groups to manage not only a mental health problem, but any situation that may arise from the transition process and to be equipped with the necessary tools to build new relationships and integrate successfully in their new communities.

## Supplementary Information

Supplementary Material 1.

## Data Availability

The data and materials are available from the corresponding author on reasonable request.

## Abbreviations

HSCL-25: Hopkins symptoms checklist
IPL-12/24: Immigration policy lab
PTSD: Post-traumatic stress disorder
COPE: Coping Orientation to Problems Experienced Inventory
ICD-10: International statistical classification of diseases and related health problems 10^th^ edition

## Data availability

No datasets were generated or analysed during the current study.
