# Supplementary material for: Navigating wellness through integration: coping strategies for depression among Syrian refugees in Norway
Source: BMC Psychol. 2024 Sep 14;12:485. doi: 10.1186/s40359-024-01987-0 (PMC11401405; doi:10.1186/s40359-024-01987-0)
Supplement: Supplementary file 1 — Supplementary Material 1. [file 40359_2024_1987_MOESM1_ESM.docx]

**Manuscript contribution**

As a result of the migration process and previous traumatic experiences, many people, especially refugees, may suffer from mental health problems such as depression. Depression can be associated with emotional and cultural changes that affect integration into the host society. When refugees have been affected by traumatic situations and mental health problems, ways of coping with a mental health problem are considered essential for integration. Recent studies suggest that a lack of integration into the host society may also contribute to poor mental health among refugee groups. As a result of the acculturation strategy of integration, non-dominant groups retain both their native culture and the host society. Successful integration of refugee groups may depend on their ability to adapt to the new cultural aspects of the community rather than losing their ancestry. Successful integration was measured in this study using several dimensions of integration: psychological, social, linguistic, economic, and navigational. Better mental health was associated with the psychological aspect of integration, which includes a sense of security or belonging in the host country. However, some believe that the psychological aspect of integration is not the only important factor. For instance, social integration, or the ability to interact with people from the host society, can also be critical. Finding one's way around the system or finding a job and being able to support oneself in the new society can also be essential to successful integration. This study examined the relationship between integration and ways of coping with depression among Syrians living in Norway. Due to the multiple aspects of the integration process, this study has provided insight into how Syrians cope with depression as they integrate into Norwegian society. The active coping mechanisms that emerged from this study indicated that specific ways of coping with depression might explain why some people might be better at certain types of coping than others. Depressed individuals may be negatively impacted if they have difficulty accessing quality mental health resources or are under increased stress due to certain aspects of the integration. Mental health problems can have serious repercussions, such as decreased physical health and increased social isolation. Due to a lack of social support and needed help, individuals may be unable to effectively implement strategies and achieve desired outcomes. This can leave depressed individuals unable to cope with their difficulties, leading to further distress and poor outcomes. Providing access to mental health services to those affected by these problems is important. Understanding how refugee groups cope with depression can help institutions provide better health services and social interactions on a day-to-day basis to help these groups better integrate into the host society. The results of this study can be used to inform policymakers and healthcare providers about the needs of refugee groups. These providers can also inform how to support better the communities where the Syrian community lives in Norway to help them better integrate into the host society. Mental health professionals can help these individuals by providing support, guidance, resources, and the necessary tools to help them adjust to their new environment.
